# Neurological Disorders and Risk of Arrhythmia

**DOI:** 10.3390/ijms22010188

**Published:** 2020-12-27

**Authors:** Joyce Bernardi, Kelly A. Aromolaran, Ademuyiwa S. Aromolaran

**Affiliations:** Biomedical Research and Translational Medicine, Masonic Medical Research Institute, 2150 Bleecker St., Utica, NY 13501, USA; jbernardi@mmri.edu (J.B.); kellyaromolaran@gmail.com (K.A.A.)

**Keywords:** mental illness, cardiovascular disease, ion channels, antipsychotic drugs

## Abstract

Neurological disorders including depression, anxiety, post-traumatic stress disorder (PTSD), schizophrenia, autism and epilepsy are associated with an increased incidence of cardiovascular disorders and susceptibility to heart failure. The underlying molecular mechanisms that link neurological disorders and adverse cardiac function are poorly understood. Further, a lack of progress is likely due to a paucity of studies that investigate the relationship between neurological disorders and cardiac electrical activity in health and disease. Therefore, there is an important need to understand the spatiotemporal behavior of neurocardiac mechanisms. This can be advanced through the identification and validation of neurological and cardiac signaling pathways that may be adversely regulated. In this review we highlight how dysfunction of the hypothalamic–pituitary–adrenal (HPA) axis, autonomic nervous system (ANS) activity and inflammation, predispose to psychiatric disorders and cardiac dysfunction. Moreover, antipsychotic and antidepressant medications increase the risk for adverse cardiac events, mostly through the block of the human ether-a-go-go-related gene (hERG), which plays a critical role in cardiac repolarization. Therefore, understanding how neurological disorders lead to adverse cardiac ion channel remodeling is likely to have significant implications for the development of effective therapeutic interventions and helps improve the rational development of targeted therapeutics with significant clinical implications.

## 1. Introduction

Psychiatric disorders are widely prevalent globally, affecting about 25–30% of patients in Europe and the United States, with anxiety disorder and depression being the most common conditions (7% and 5% respectively) [1]. Cardiovascular disorders (CVDs) are the leading cause of death in the general population, but also among patients with neurological diseases [2], suggesting a link between these two populations.

In general, a complex set of behavioral and psychosocial aspects are mediators for increased CVD risk, including smoking, alcohol and substance abuse, poor diet and reduced physical activity that can lead to obesity, non-adherence to medications and sleep disorders, anger and hostility, social isolation and low socioeconomic status [3,4,5]. These CVD risk factors are significantly present among subjects with mental illness, resulting in an additive effect over the disease-related biological risk factors [6] that these patients have for CVD. Notably, drug therapies for the treatment of mental disorders predispose to a variety of physical illnesses (obesity, diabetes, thyroid disorders, gastrointestinal, respiratory and renal diseases, etc.), including CVD and arrhythmias [7,8]. All-cause mortality in general, and cardiac mortality in particular, is higher in antipsychotic users compared to nonusers [9].

Therefore, there is an urgent need for management strategies to reduce the CVD risk in this population group. A holistic understanding of the molecular mechanisms that underlie biological stressors is important in defining psychological and physical outcomes that determine vulnerability to disease conditions, and in particular CVD [10]. This is also valid for CVD patients, in which psychological and psychiatric problems (such as depression and anxiety) that may arise following major cardiac events are often under-reported and undertreated. Therefore, a prompt identification and treatment of potential psychological conditions could help reduce the risk of further cardiac events and improve the outcome in cardiac patients.

In this review we discuss existing knowledge of the intimate and delicate interaction between neurological disorders and CVD, taking into consideration common and distinct pathological mechanisms. In particular, we discuss the potential involvement of pathological ion channel modulation in the etiology of neurological disorders with significant implications for CVD and ultimately arrhythmias. Our hope is that this review will be of great interest to a wide range of the scientific community and more specifically neurology and cardiology research investigators. In this context our goal is to further highlight unacknowledged common and unique molecular mechanisms of neurological channelopathies and cardiomyopathies that merits significant investigation.

## 2. Psychiatric Disorders and Cardiovascular Diseases

A bidirectional relationship between mental illness and CVD is known to exist. Among mental illnesses, depression, post-traumatic stress disorder (PTSD), anxiety, schizophrenia and autism are the most commonly studied due to their crucial predisposition to adverse cardiac events [2,11,12,13,14,15]. For example, depression is a mood disorder that varies from mild to major depressive symptoms and is characterized by sadness, pervasive low mood and loss of interest (anhedonia) lasting for 2 weeks or more [2,11]. Depression and cardiovascular disorders are closely related. CVD can cause depressive symptoms, and the prevalence of depression in patients with CVD is 3 times higher than in the general population [16]. Furthermore, depression has been reported to be an independent risk factor for cardiac events [17], increasing the incidence of CVD in previously healthy people [18].

Depression and anxiety are interlinked pathologies, but the associated mechanisms are unknown or poorly understood. Notably, patients with high levels of anxiety have an increased risk for sudden cardiac death (SCD) [13,19,20]. Indeed, hyperventilation, that may occur during an acute panic/anxiety attack, can induce coronary artery spasm [21], which in turn may eventually lead to myocardial ischemia and fatal ventricular arrhythmias [22,23].

Depressive and anxiety disorders have a high comorbidity and share symptoms with PTSD, a disease state defined by trauma and stressor-related diseases that may develop after a major traumatic event (including combat, sexual assault, etc.). Further, intrusive thoughts, negative cognitions and mood, avoidance and hyperarousal are associated with PTSD and this, in turn, leads to severe distress. For example, clinically relevant studies in the Veterans population have highlighted the association between PTSD and CVD, with PTSD patients having double the risk of developing adverse cardiac events [24,25,26,27,28]. Moreover, experiencing a life-threating illness, including a major cardiac event, can elicit PTSD, and the persistence of PTSD symptoms can increase the likelihood of developing recurrent CVD [29,30,31].

Schizophrenia is another psychiatric disorder significantly associated with augmented risk for CVD [14,32]. Schizophrenia is defined by the presence of two or more characteristic symptoms, including hallucinations, disorganized speech and delusions. Patients with schizophrenia are likely to have a 10 years lower life-expectancy compared to the general population, and this dramatic reduction is underscored by a high incidence of suicide and an elevated CVD risk [33].

Autism spectrum disorder (ASD) is a neurodevelopmental disorder that is characterized by restricted interests, repetitive behaviors and difficulties in communication and social interaction. ASD is commonly comorbid with other psychiatric disorders (depression and anxiety), and also with epilepsy, suggesting the existence of shared biological mechanisms between these conditions. Congenital heart diseases (CHDs) such as atrial and ventricular septal defects have been associated with an increased risk of developing ASD [34] and epilepsy [35]. While the exact cause is unknown, studies have suggested that there could be common genetic links [36], environmental causes or it could be due to surgeries or other clinical outcomes due to CHD. For example, increased seizures for CHD patients, in general and particularly after surgery, lead to deficits in neural development that might be due to cerebral hypoperfusion [37,38], and further reinforces an important physiological interplay between these disease pathologies. Furthermore, ASD patients are more likely to have hyperlipidemia, which is a known risk factor for diabetes, obesity and CVD [15]. Others and we have demonstrated that pathological levels of the saturated free fatty acid, palmitic acid, led to adverse remodeling of major cardiac ion channels in distinct animal models [39,40,41,42]. These findings suggest a higher likelihood of experiencing a fatal arrhythmia event and ultimately the transition to heart failure and sudden cardiac death in ASD patients with confounding hypercholesterolemia and/or hypertriglyceridemia. Future studies of the mechanisms of the neurological–cardiac axis that include patients with lipid metabolism disorders are likely to provide novel and additional insights that will improve knowledge of vulnerability of ASD patients to metabolic disorders and ultimately cardiac dysfunction.

Antipsychotic and antiepileptic medications have been reported to have a range of cardiac side-effects, including orthostatic hypotension [43], cardiomyopathy [44], QT prolongation [45] and increased risk for SCD [9]. Moreover, antidepressant drugs have been associated with adverse cardiac effects: the selective serotonin reuptake inhibitors (SSRIs) and particularly the tricyclic antidepressants are known to cause prolongation of the heart rate corrected QT interval (QT_c_) on an ECG and predispose to ventricular arrhythmias [46,47]. These cardiotoxic effects of psychiatric disorder therapeutics are of particular importance in patients with an underlying CVD.

Neurological conditions, including subarachnoid hemorrhage, can also be associated with cardiac dysfunction. In this context the Krzych lab demonstrated that the neurocardiogenic injury that follows a subarachnoid hemorrhage is characterized by ST-segment elevation and QT_c_ prolongation on the ECG, moderate elevation in Troponin C levels and myocardial necrosis [48]. These clinical presentations are reminiscent of Takotsubo cardiomyopathy a cardiac condition that develops in response to severe psychological distress, or an intense emotional or stressful experience [49]. A catecholamine-induced toxicity in cardiomyocytes has been identified as a common pathological mechanism between the two conditions, and further highlights a critical link and/or interplay between cellular functions of the brain and heart.

## 3. Common Molecular Mechanisms of Cardiovascular Disorders Acquired in Mental Disorders

**Figure 1:**
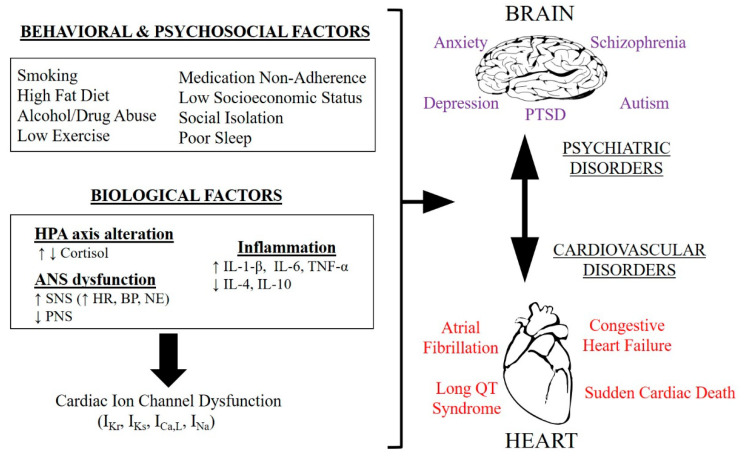
Functional interaction between brain and heart disorders. Scheme of the behavioral, psychosocial and biological risk factors shared among psychiatric disorders that are involved in the increased incidence of cardiovascular diseases. HPA axis = Hypothalamic–Pituitary–Adrenal axis, ANS = Autonomic Nervous System, SNS = Sympathetic Nervous System, PNS = Parasympathetic Nervous System, HR = Heart Rate, BP = Blood Pressure, NE = Norepinephrine, TNF-α = Tumor Necrosis Factor Alpha, PTSD = Post-Traumatic Stress Disorder, *I_Kr_* = Rapid Delayed Rectifier Potassium Current, *I_Ks_* = Slow Delayed Rectifier Potassium Current, *I_Ca,L_* = L-Type Calcium Current, *I_Na_* = Sodium Current, ↑ increased, ↓ decreased.

Psychiatric diseases share common biological, behavioral and psychosocial risk factors that increase the likelihood of developing CVD [50]. Dysfunction of the hypothalamic–pituitary–adrenal (HPA) axis and autonomic nervous system (ANS) activity, inflammation and oxidative stress are key cellular mechanisms that play an important role in the development of mental conditions [51], as summarized in Figure 1.

The HPA axis is responsible for the release of different neuropeptides and hormones, including cortisol, crucial for the physiological response to stress and the subsequent regulation of individual or multiple combinations of homeostatic processes that includes emotional, metabolic, cardiovascular and immune mechanisms. Prolonged, excessive or insufficient activity of the HPA axis, particularly in conjunction with repetitive exposure to stress, may result in the development of psychiatric disorders, such as depression, anxiety disorders, PTSD, schizophrenia and autism [52,53]. Similarly, HPA axis hyperactivity has been associated with increased CVD risk and mortality [54,55].

The stress response system is defined by both the HPA axis, and the ANS. The ANS is composed of two branches: the sympathetic nervous system (SNS) and the parasympathetic nervous system (PNS). In response to stressful situations, the SNS is the dominant mechanism exemplified by the activation of the fight-or-flight response, which leads to catecholamine (noradrenaline and norepinephrine) release. Furthermore, ANS activation leads to physiological changes, which include an increase in heart rate (HR) and blood pressure. SNS hyperactivation coupled with blunted PNS activity can play a role in different psychiatric disorders [56,57]. The resulting ANS dysfunction is characterized by high levels of circulating catecholamines (primarily norepinephrine), higher HR at rest, low HR variability (HRV), baroreflex dysfunction and increased QT_c_ variability [56,58,59,60]. The relative functional contributions of the different altered neurological signaling mechanisms to cardiac dysfunction are unknown. Therefore, studies that provide molecular mechanisms of common or unique signaling pathways are likely to provide mechanistic insights that will improve our understanding of the delicate link between neurological disorders and vulnerability to arrhythmogenesis and heart failure.

There is evidence that a dysregulated stress response system that occurs in mental disorders, leads to hyperinflammation, which is an independent risk factor for CVD [61]. Furthermore “sterile inflammation”, a process that is not due to a pathogen, but can result after chronic stress, is evident in different mental disorders. As a consequence of altered neurohumoral function, the pathological levels of proinflammatory cytokines including interleukin (IL)-6, IL-1β and tumor necrosis factor alpha (TNF-α) can be triggered, especially in microglia and astrocytes. In fact, an increased proinflammatory setting is reported in patients with depression, schizophrenia, PTSD, anxiety disorder and ASD [62,63,64,65,66]. Inflammation is a shared mechanism between psychiatric disorders and CVD. Proinflammatory cytokines like IL-6, IL-1β and TNFα are involved in the progression of chronic heart failure through remodeling and increased fibrosis [67], and these alterations can further lead to susceptibility of ventricular tissue to arrhythmias. Additionally, cytokines have been shown to modulate different cardiac ion channels and this interaction can have important effects on action potential (AP) and QT duration [68], having either protective or pathological consequences, such as promotion of arrhythmias [69]. IL-1β and IL-6 increase the L-type calcium current (*I*_CaL_), while TNF-α decreases both the transient outward (*I*_to_) and the rapid delayed rectifier (*I*_Kr_) potassium currents, all leading to an increase of AP duration (APD) in cardiomyocytes [70,71,72,73]. IL-1 and TNF-α have been positively associated with QT_c_ prolongation [74,75], while IL-6 and IL-8 have been correlated with SCD [76,77]. There is also a crosstalk between inflammation and ANS: stimulation of the SNS has been shown to increase the level of proinflammatory cytokines, while PNS activation seems to promote the reduction of such cytokines; at the same time these pro-inflammatory cytokines modulate ANS action on the heart, mainly acting on HRV [69].

Comprehensive behavioral animal studies, in response to specific (acute vs. chronic) stressors have revealed subjective outcomes, defined by distinctive active and/or passive coping mechanism. For example, an active coping mechanism would result in a fight-or-flight response, while a passive coping response is characterized by immobility, avoidance and withdrawal. The prevalence of one approach over the other can then influence the psychological and metabolic state of the organism. Indeed, stress-susceptible animals usually display altered HPA axis function, increased sympathetic activity and reduced parasympathetic tone, with associated pathological elevations of catecholamines, high blood pressure, increased resting HR, decreased HRV [78] and a higher secretion of proinflammatory cytokines. This finding would suggest that stress-resistant animals are likely to display an opposing phenotype that includes a normal representation of anti-inflammatory cytokines, including IL-4 and IL-10 [79,80,81]. Moreover, social defeat and isolation have been shown to have cardiac consequences (decreased HRV and hypertrophy) in animal experiments, and aggressive animals were more vulnerable to heart disease [78], suggesting that coping mechanisms are related with risk for CVD.

## 4. Evidence for a Potential Role for Ion Channels Linking Neuropsychiatric Disorders and CVD

Ion channels are widely distributed in the brain and heart. Both neurons and cardiomyocytes are excitable cells; therefore, the biophysical properties and regulation of ion channels are important for their functional activity. In some cases, a connection between cardiac and neuronal ion channel dysfunction can be present, resulting in clinical conditions with both psychiatric and cardiac phenotypes. This is highlighted in Timothy syndrome (TS) or long QT syndrome type 8 (LQT8).

TS is a multisystemic syndrome that is caused by congenital or inherited mutations in the CACNA1C gene, which encodes for the alpha 1C subunit of the L-type calcium channel (Cav1.2) and contributes prominently to normal cardiac repolarization and neurological functions including synaptic plasticity and long-term potentiation. TS is characterized by a combination of QTc prolongation, syndactyly and autism. Typical TS (type 1) results from a recurrent de novo mutation, G406R in CACNA1C exon 8A, while atypical TS (type 2) characterized by the lack of syndactyly phenotype, is caused by G406R and G402S mutations in the alternatively spliced exon 8. A de novo CACNA1C mutation, the G402S substitution in exon 8, is associated exclusively with a cardiac specific phenotype (including LQTS and cardiac arrest), with no signs of multiorgan disease manifestations of classical TS [82]. Conversely, the A1473G mutation can induce additional central and peripheral neurological symptoms (stroke, seizure, cortical blindness and development delay) in LQT8 patients [83], and of particular note, a novel A1024G mutation has been reported in patients with extracardiac symptoms but no QT prolongation [84]. The phenotypical variability of this disease has been attributed to the fact that distinct missense mutations may differently affect excitable and non-excitable cells [85].

Mutations in the KCNJ2 gene, that encodes for the Kir2.1 channel subunit also led to channelopathies defined by defects in both cardiac and neuronal mechanisms. Kir2.1 generates the inward-rectifier K current *I*_K1_, which is abundant in the brain and human cardiac and skeletal muscles [86]. KCNJ2 mutations (including T192A and R67W) that lead to a loss-of-function phenotype have been associated with Andersen–Tawil syndrome (or LQT7), a genetic condition characterized by QTc interval prolongation, muscle weakness and paralysis, mood disorders and seizure [87]. Gain-of-function mutations in the KCNJ2 gene (D172N, M301K, E299V and K346T) are instead, linked to QT shortening and increased risk for SCD. Of particular note, a novel p.Phe58Ser missense mutation has been shown to lead to both short QT syndrome type 3 (SQT3) and autism [88,89], with the gain-of-function effect of this mutation consistent with the altered neuronal excitability found in ASD. Distinguishing between mutations that lead to cardiac effects or both cardiac and neuronal effects is likely to provide crucial insights that will inform development of targeted interventions that will treat neurological disorders and prevent off-target cardiac effects.

This connection is also true for NOS1AP (nitric oxide synthase 1 adaptor protein), an adaptor protein that plays a crucial role in both cardiac and neuronal calcium handling mechanisms and ion channel regulation [90]. NOS1AP is important for the activity of the neuronal isoform of NOS (nNOS or NOS1), which in turn modulates different processes but mainly the S-nitrosylation of Cav1.2 channels. Single-nucleotide polymorphisms (SNPs), such as the minor NOS1AP rs16847548, have been associated with decreased expression of NOS1AP resulting in upregulation of *I*_CaL_, with increased risk for arrhythmias [91]. NOS1AP has been identified as a modifier gene for LQTS, with SNPs (rs4657139 and rs16847548) associated with QT prolongation in the general population and with an increased risk for SCD in LQT1 patients [92], but it is also a susceptibility locus for schizophrenia (SNPs rs1415263, rs4145621 and rs2661818) [93]. This evidence further reinforces a role for NOS1AP as a key candidate for the crucial mechanistic link that underlies arrhythmogenesis in neuronal disorders.

The most prevalent types of LQTS are caused by mutations in three cardiac channels: KCNQ1, KCNH2 and SCN5A, which have been also associated with epilepsy and sudden unexpected death in epilepsy (SUDEP) [94]. SUDEP is characterized by the absence of any cause of death at post-mortem evaluation, and therefore an arrhythmogenic mechanism is generally suggested. The association between LQTS genes and epilepsy has been complicated by the fact that epileptic seizures can be misinterpreted as syncope due to cardiac arrhythmias in LQTS patients. About one out of five LQT1 patients experiences a seizure phenotype, supporting the idea that KCNQ1 mutations can increase the susceptibility for epileptic seizure [95]. Indeed, a non-synonymous heterozygous missense pathogenic mutation (p.L273F) in exon 6 of the KCNQ1 gene (encoding for the Kv7.1 channel) has been identified in a LQT1 family with recurrent epilepsy [96], further suggesting the neurocardiac effect of this gene.

Mutations and SNPs in KCNH2 have also been linked with epilepsy and SUDEP, and retrospective studies have found that a history of seizures was more common among LQT2 patients compared to the other LQT subtypes [97], and further reinforces an important contribution of KCNH2 defects to epilepsy predisposition. Similarly, a p.W1095X (or p.Trp1095STOP) missense mutation in the SCN5A gene has been identified in subjects showing both Brugada symptoms and epilepsy [98]; and another SCN5A mutation (Pro1090Leu) previously associated with SCD and LQTS, has been found in a SUDEP victim [94].

Growing evidence supports the involvement of ion channels that play a significant role in cardiac function to also play a role in the susceptibility or pathogenesis of psychiatric disorders, but the extension of their contribution may be largely variable. Ion channel mutations can either be the only cause, as occurs in TS, or most commonly, their association with psychiatric disorders is more complex, and can be the result of haplotype effects. SNPs in Ca channel (CACNA1C and CACNB2) genes have been identified as a susceptibility factor for bipolar disorder, schizophrenia and major depression [99,100], suggesting that altered functional expression of voltage-gated Ca channels is an important shared factor in psychiatric disorders. This association is further supported by functional magnetic resonance imaging studies that have correlated the presence of the risk-associated CACNA1C SNP (rs1006737) and activation of brain circuitries that are characteristic of patients with mental illness [101].

Mutations in Na channel genes have been extensively linked to epilepsy [102]; however, Okamura’s group have reported a link between a novel SCN1A mutation (V1366I) in a Japanese family with different mental illness phenotypes including Asperger syndrome, ASD and panic disorder [103]. Deletion in neuronal specific SCN2A and SCN3A genes have been associated with autism [104] and different forms of epilepsy [105], while loss-of-function mutations in the SCN1A gene are responsible for the comorbidity of psychiatric disorders with epilepsy [106], suggesting that Na channels may be functionally important for emotional and cognitive responses.

K channels including the KCNQ family play a role in the etiology of psychiatric diseases [107]. For example, KCNQ2 and KCNQ3 genes have been identified as putative risk factors for bipolar disorders [108,109], and in particular, specific KCNQ2 variants (with a shorter C-terminal) are associated with suppressed channel activity, concordant with an effect on neuronal hyperexcitability, characteristic of a manic state [110]. The human ether-à-go-go related (hERG) gene, encoding for the Kv11.1 channel, has also been linked with schizophrenia [111]. Due to the pivotal role for the hERG channel in cardiac repolarization [112], we will discuss its significance in the pathological outcome of brain disorders in subsequent sections below.

Distinct and targeted pharmacological interventions for treatment of psychiatric disorders are known to interact with ion channels, suggesting a potential involvement of off-target cardiac effects of pharmacological treatments designed to treat neurological disorders. Typical antidepressant drugs such as SSRIs have been shown to have an inhibitory effect on Na channel function [113], and more importantly, an elevation in whole-body residual Na concentration has been observed in patients with depression and particularly those in a manic state, which is an important signature of bipolar disorder and schizophrenia [114]. Similarly, lithium, a well-known pharmacologic, which is used for the treatment of bipolar disorders, has been shown to block cardiac Na channels [115], leading to the unmasking of adverse ECG abnormalities that are characteristic of the Brugada syndrome [116]. Furthermore, Ca channel blockers, such as verapamil and nicardipine, generally used in the treatment of angina, hypertension and supraventricular arrhythmias, have also been shown to display antipsychotic effects [117].

Moreover, antiepileptic drugs that target ion channels are generally prescribed as an effective treatment option for psychiatric disorders (schizophrenia, bipolar and anxiety disorders). For example, a variety of non-selective Na channel blockers initially designed as antiepileptics have also been used as effective therapeutics as mood stabilizers and antidepressants [118], while K channel enhancers or activators act as antipsychotics and/or anticonvulsants [119,120]. This broad therapeutic effect of antiepileptic drugs further reinforces the shared pathophysiology that exist between epilepsy and psychiatric disorders and suggests adverse ion channel modulation as a key mechanism linking these pathologies [121].

## 5. hERG and *I_Kr_* Channel Modulation as an Important Pathological Link between Neuronal Disorders and Vulnerability to Arrhythmias

In human heart, hERG1a/1b subunits coassemble to generate *I*_Kr_ [112], which is important for normal repolarization [122]. Pathological depression of cardiac *I*_Kr_, either due to inherited mutations in hERG or drug-induced, results in a delayed repolarization leading to the prolongation of the QT_c_ interval, a disease state that predisposes to fatal arrhythmias such as Torsades des Pointes [123,124], which affects the young and also the old and ultimately the transition to heart failure and sudden cardiac death. hERG channel subunits have an important function in other cell types including neuronal and muscle cells (neuroblastoma cells, neuroendocrine cells and smooth muscle fibers of gastrointestinal tract) [122]. hERG1 is predominantly expressed in the heart, although in the brain, currents through hERG1 channel subunits play an important role in neuronal excitability and firing, while hERG2 and hERG3 are exclusively expressed in the brain [125,126,127].

Similarly, hERG channels expressed in dopamine neurons have been shown to regulate neuronal excitability [126], suggesting that similar, if not identical, hERG channel biophysical properties may exist in both cardiomyocytes and neuronal cells. This notion is reinforced by the demonstration that antipsychotic drugs that display antidopaminergic properties have been shown to interact with hERG channels [128] and this non-selective effect has been suggested to contribute to the therapeutic activity [126] and possible cardiac off-target effects. In most cases of targeted modification of cardiac hERG channel function, major underlying molecular mechanisms include defects in channel gating and trafficking [112,129,130]. Therefore, it would be interesting to know whether similar mechanisms underlie neuronal hERG channel functional properties.

Huffacker and others [131] have previously reported the expression of a primate- and brain-specific hERG channel isoform KCNH2-3.1, whose encoding gene is localized in close proximity to the risk-associated SNPs for schizophrenia. This novel isoform has been identified in specific brain regions of individuals affected with schizophrenia and found to harbor schizophrenia susceptibility alleles [131]. In patients with schizophrenia, these risk alleles have not been associated with changes in the functional expression of KCNH2-1a and KCNH2-1b, but the ratio between KCNH2-3.1 and KCNH2-1a isoform was 2.5-fold higher compared to healthy subjects, suggesting that overexpression of the KCNH2-3.1 isoform could have a prominent role in the pathogenesis of this psychiatric disease [131]. Electrophysiological assays in both HEK cells and rat primary cortical transfected neurons have revealed that currents generated by KCNH2-3.1 subunits display rapid deactivation kinetics, possibly due to the lack of the N-terminal domain crucial for slow deactivation. This biophysical property may underlie increases in spike frequency and the switch from adapting to non-adapting firing patterns in cortical neurons [131]. Therefore, its enhanced expression is associated with increased neuronal excitability, matching the results observed in brain areas of psychiatric subjects [111,131].

Subjects with the risk genotype associated with higher expression of the KCNH2-3.1 isoform seem to have an increased response to antipsychotic drugs, emphasizing this variant as a possible therapeutic target [132]. The brain specific expression of KCNH2-3.1 could be a highly beneficial target for future treatment options for neurological disorders but without the off-target cardiac effects that are likely to predispose to arrhythmias possibly through adverse modulation of hERG channel function.

## 6. Conclusions and Future Directions

It is becoming increasingly clear that distinct biological, behavioral and psychosocial factors mediate the physiological link between mental illnesses and the increased risk of CVD. Therapeutic strategies are also known to increase the risk for CVD. In fact, antidepressants that target serotonin or norepinephrine reuptake, or antipsychotic drugs blocking dopamine receptors, are the most commonly used therapeutics in clinical interventions [133], and several of these drugs are known to be proarrhythmic, mainly due to their effect of cardiac hERG channels blockade.

Anti-inflammatory treatment strategies in neurological diseases have shown promising results mostly by limiting depressive symptoms [134]. Therefore, a combination of therapeutics including those that target hyperinflammatory cellular signaling pathways, could help to improve outcomes in patients. Moreover, considering the elevated proinflammatory profile found in different psychiatric disorders and the proarrhythmic effect of specific cytokines, therapies that aim at lowering inflammation could both improve psychiatric symptoms and reduce the risk for CVD and arrhythmias.

The majority of studies on the association between arrhythmias and psychiatric disorders describe evidence for ventricular arrhythmias (LQT) but less is known for other forms of arrhythmias. Few trials have attempted to investigate the prevalence of atrial fibrillation in mental disorders, but found that panic disorder and likely anxiety, are associated with increased incidence of atrial fibrillation [135,136]. Additional studies assessing the occurrence of other types of arrhythmias in psychiatric disorders could provide further insight into the pathological mechanisms of such diseases.

Further it is known that mental diseases are generally associated with behavioral and/or lifestyle changes including smoking, poor diet, reduced physical activity, alcohol and substance abuse and non-adherence to medications. Therefore, coupling therapeutics with clinical interventions that limit significant changes in individual or multiple combinations of life-style behaviors is likely to reduce the risk of developing cardiovascular diseases that predispose to heart failure.

Finally, the involvement of ion channels in the etiopathology of psychiatric disorders may support the evaluation of alternative targets for the development of pharmacological strategies. The evidence that subjects with a particular neuronal specific hERG isoform (KCNH2-3.1) associated with schizophrenia show a higher responsiveness to antipsychotic drugs and is a relevant example of ion channels as a therapeutic target. Therefore, a comprehensive investigation of the functional interplay between cardiac and neuronal ion channels in the pathogenesis of mental illness and CVD is likely to be rewarded by mechanism-based insights that will help to improve the clinical limitations of existing therapeutic and behavioral interventions in patients.

## Data Availability

Not applicable.

## Abbreviations

ANS	Autonomic Nervous System
ASD	Autism Spectrum Disorder
CHD	Congenital Heart Diseases
CVD	Cardiovascular Disorders
hERG	Human Ether-A-Go-Go-Related Gene
HPA axis	Hypothalamic–Pituitary–Adrenal Axis
HRV	Heart Rate Variability
*I*_Ca,L_	L-Type Calcium Current
*I*_Kr_	Rapid Delayed Rectifier Potassium Current
*I*_to_	Transient Outward Potassium Current
IL-6	Interleukin 6
IL-1β	Interleukin 1β
LQTS	Long QT Syndrome
PNS	Parasympathetic Nervous System
PTSD	Post-Traumatic Stress Disorder
QTc	Corrected QT interval
SCD	Sudden Cardiac Death
SNP	Single Nucleotide Polymorphisms
SNS	Sympathetic Nervous System
SQTS	Short QT Syndrome
SSRI	Selective Serotonin Reuptake Inhibitors
TNF-α	Tumor Necrosis Factor Alpha
TS	Timothy Syndrome
